# Phonemic fluency in post-ICU patients after severe COVID-19 infection: The role of cognitive reserve

**DOI:** 10.1192/j.eurpsy.2022.954

**Published:** 2022-09-01

**Authors:** X. Segú, M. Primé Tous, M. Sanchez, F. Valdesoiro, A. Rodriguez, I. Martín, A. Costas

**Affiliations:** Hospital Clínic de Barcelona, Department Of Psychiatry And Psychology, Barcelona, Spain

**Keywords:** cognitive impairment, Neurological manifestations, Covid-19, Cognitive reserve

## Abstract

**Introduction:**

Cognitive function may be impaired in COVID-19 patients, especially in executive functions such as phonemic fluency. Among risk factors, inflammation during hospitalization is related with worse cognitive performance in the long term. On the other side, it has been shown that cognitive reserve (CR) protects against cognitive impairment associated with brain damage, psychiatric disorders and neurodegenerative diseases.

**Objectives:**

Our aim is to study the protective role of cognitive reserve in phonemic fluency to inflammation after SARS-CoV-2 infection.

**Methods:**

We enrolled a cohort of 102 severe SARS-CoV-2 survivors after Intensive Care Unit (ICU) discharge and 58 agreed to participate in this 6-month follow-up study. Patients with previously known cognitive impairment were excluded. Demographic, clinical and laboratory data were collected. To assess the phonemic fluency, we used the Controlled Oral Word Association Test (COWAT) controlling the effects of age and education. Inflammation was recorded according to the number of days with high CRP. ANCOVA analyses were used to test the effect of interaction between medical variables and cognitive reserve on phonemic fluency.

**Results:**

The COVID-19 inflammation interacted with CR in phonemic fluency (F= 6.47, p= 0.01), with worse performance in patients with low CR (mean 16.7 (10.2-23.3)) than those with high CR (mean 37.7 (34.3-41.2)) in function of number of days with high PCR during ICU stay.

**Conclusions:**

The role of the cognitive reserve is important to reduce the cognitive impairment related with COVID-19 inflammation in post-ICU patients.

**Disclosure:**

No significant relationships.

